# Risk clusters of COVID-19 transmission in northeastern Brazil: prospective space–time modelling

**DOI:** 10.1017/S0950268820001843

**Published:** 2020-08-24

**Authors:** D. S. Gomes, L. A. Andrade, C. J. N. Ribeiro, M. V. S. Peixoto, S. V. M. A. Lima, A. M. Duque, T. M. Cirilo, M. A. O. Góes, A. G. C. F. Lima, M. B. Santos, K. C. G. M. Araújo, A. D. Santos

**Affiliations:** 1 Universidade Federal de Sergipe, Programa de Pós-graduação em Biologia Parasitária, Aracaju, Sergipe, Brazil; 2 Universidade Federal de Sergipe, Núcleo de Investigação em Saúde Coletiva, Aracaju, Sergipe, Brazil; 3 Universidade Federal de Sergipe, Programa de Pós-graduação em Enfermagem, Aracaju, Sergipe, Brazil; 4 Instituto Federal de Educação, Ciência e Tecnologia de Sergipe, São Cristóvão, Sergipe, Brazil; 5 Departamento de Fonoaudiologia, Universidade Federal de Sergipe, Aracaju, Sergipe, Brazil; 6 Departamento de Enfermagem, Universidade Federal de Sergipe, Lagarto, Sergipe, Brazil; 7 Departamento de Terapia Ocupacional, Universidade Federal de Sergipe, Lagarto, Sergipe, Brazil; 8 Secretaria Estadual de Saúde de Sergipe, Aracaju, Sergipe, Brazil; 9 Departamento de Medicina, Universidade Federal de Sergipe, Aracaju, Sergipe, Brazil; 10 Departamento de Morfologia, Universidade Federal de Sergipe, Aracaju, Sergipe, Brazil

**Keywords:** COVID-19, disease surveillance, pandemic, space–time cluster, spatial analysis

## Abstract

This study aimed to analyse the trend and spatial–temporal clusters of risk of transmission of COVID-19 in northeastern Brazil. We conducted an ecological study using spatial and temporal trend analysis. All confirmed cases of COVID-19 in the Northeast region of Brazil were included, from 7 March to 22 May 2020. We used the segmented log-linear regression model to assess time trends, and the local empirical Bayesian estimator, the global and local Moran indexes for spatial analysis. The prospective space–time scan statistic was performed using the Poisson probability distribution model. There were 113 951 confirmed cases of COVID-19. The average incidence rate was 199.73 cases/100 000 inhabitants. We observed an increasing trend in the incidence rate in all states. Spatial autocorrelation was reported in metropolitan areas, and 178 municipalities were considered a priority, especially in the states of Ceará and Maranhão. We identified 11 spatiotemporal clusters of COVID-19 cases; the primary cluster included 70 municipalities from Ceará state. COVID-19 epidemic is increasing rapidly throughout the Northeast region of Brazil, with dispersion towards countryside. It was identified high risk clusters for COVID-19, especially in the coastal side.

## Introduction

The world has been facing an international public health emergency caused by the severe acute respiratory syndrome coronavirus 2 (SARS-CoV-2), termed as coronavirus disease 2019 (COVID-19) [1]. The disease was firstly reported in the city of Wuhan (China) at the end of December 2019 and was declared a pandemic by the World Health Organisation in March 2020 [2]. There were more than 17 million cases and over 650 000 deaths by COVID-19 confirmed worldwide [1]. Notably, the pandemic has been challenging for health systems and governments, as the extent of the social and economic impacts of the pandemic is still uncertain [3].

Following the disease's dynamics and the exponential growth of the number of cases, several studies have been reported [2–5]. Particularly, those that perform temporal and spatial analyses of COVID-19 have demonstrated the impact of morbidity, mortality and global geographical dissemination of the disease in the world. The use of aggregate spatial data allows to map the patterns of the rapid progression of the disease and to support decision-making in the allocation of resources for the prevention and control of COVID-19 in priority areas [3–6].

In this context, a study that used spatial analysis techniques, conducted in China, found that SARS-CoV-2 infection was spatially dependent and spread mainly from Hubei province, in Central China, to the surrounding areas [7]. Additionally, the spatial distribution of cases and mortality by COVID-19 is heterogeneous across regions, especially in those with socioeconomic disparities [8, 9].

This uneven geographic distribution has been observed in several regions of the United States. The disease has disproportionately affected populations in situations of social vulnerability, as observed in poorer communities from Chicago and New York. The inadequate effects of COVID-19 reflect the social inequities that existed prior to the current health crisis [9].

Recently, Brazil has become the epicentre of the epidemic in Latin America and ranks second in the world in the total number of cases (behind only the USA) [10]. COVID-19's first case was confirmed on 26 February 2020 and the country currently has more than half a million cases and about 30 000 deaths. Among Brazilian regions, the Northeast ranks second with the highest number of cases [11]. Additionally, Brazil is still marked by great social and human development inequalities, especially in the Northeast region [12]. This highlights the need for scientific research on the epidemiology and spatial distribution of COVID-19 in the municipalities of this region.

Notably, studies of spatial and temporal patterns help to elucidate the mechanisms of disease spread in the population and to identify factors associated with heterogeneous geographic distribution [3, 6]. Similarly, prospective space–time analysis is required to monitor outbreaks, as it allows the detection of active, emerging clusters and the relative risk (RR) for each affected site during the epidemic [13]. Therefore, considering the current situation of COVID-19 in the regions of the country, the study aimed to analyse the trend and spatial–temporal clusters of transmission risk of COVID-19 in northeastern Brazil, defining priority areas for surveillance actions and more effective disease control in the states.

## Materials and methods

### Study design

We conducted an ecological study with techniques of spatial analysis and temporal trend. All confirmed cases of COVID-19 in the Northeast region of Brazil were included, from 7 March to 22 May 2020 (divided into 12 epidemiological weeks). The units of analysis were the nine states (federative unit) in the region and its 1794 municipalities. Data were collected daily regarding the municipality of residence of confirmed cases of COVID-19. We excluded 1519 cases with no data of county location [14].

### Study area description

**Fig. 1.**
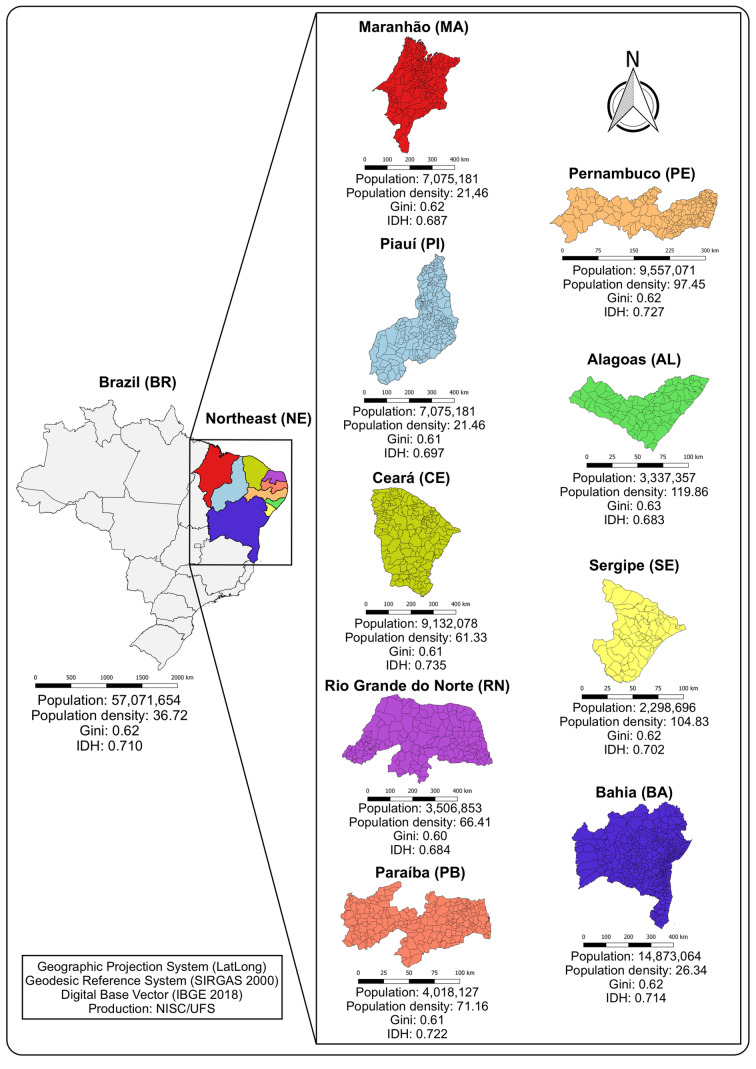
Study location.

Brazil occupies a territorial area of 8.51 million km^2^, which is equivalent to almost 50% of South America territory and has a total population of 210.1 million inhabitants [15]. It is the country with the fifth largest territorial area on the planet and the sixth largest population, with a demographic density of 24.47 inhabitants per km^2^ [15, 16]. The Northeast region (latitude: 01°02′30″N/18°20′07″S; longitude: 34°47′30″E/48°45′24″W) is one of the five Brazilian regions and the one with the largest number of federative units (nine) (Fig. 1). This region has the third largest territorial area in Brazil (155 291 744 km^2^) and a population of 57 071 654 inhabitants, which corresponds to about 30% of the Brazilian population. The highest population density occurs in the coastal strip, where most state capitals are located [16]. The Northeast region of Brazil also presents the lowest human development index in Brazil (HDI = 0.663) [17].

### Variables and measures

The variables analysed in this study were:

- New cases of COVID-19 in the 1794 municipalities of Northeast region of Brazil. The calculation was based on subtracting the previous day's count (*n_t_*) from the previous day (*n_t_*_−1_);
- Weekly incidence rates for states, metropolitan (MA) and inland areas were calculated per 100 000 inhabitants. For the calculation, we used the number of confirmed cases of COVID-19 per week in each state, in the metropolitan and inland areas, as the numerator and the corresponding populations as the denominator;
- COVID-19 incidence rates of municipalities were calculated per 100 000 inhabitants. For the calculation, the number of accumulated cases of COVID-19 in each municipality was used as the numerator and the corresponding current population as the denominator.

### Time trend analysis

To assess the weekly time trend of cases by COVID-19, we performed data analysis using the segmented log-linear regression model. The incidence rates of COVID-19 were considered dependent variables and the epidemiological weeks were the independent variables. The Monte Carlo permutation test was used to select the best model for inflection points, applying 999 permutations and considering the highest residue determination coefficient (*R*^2^). To describe and quantify the time trends, we calculated the weekly percentage increments (adapted from annual percent changes; APC) [18] and their respective confidence intervals (95% CI). Once more than one significant inflection was detected during the study period, and the average annual percentage changes (AAPCs) were calculated. Time trends were considered statistically significant when APCs had a *P*-value <0.05 and their 95% CI did not include a zero value. A positive and significant APC value indicates an increasing trend; a negative and significant APC indicates a decreasing trend and non-significant trends are described stable, regardless of APC values [19].

### Spatial cluster analysis

The raw data rates were smoothed by the local empirical Bayesian estimator [20] to minimise the instability caused by the random fluctuation of the cases. Rate smoothing was done by applying weighted averages, resulting in a second adjusted rate. The crude and smoothed incidence rates were represented in thematic maps stratified into five categories of equal intervals: (a) 0 (without a record of cases or not specified), (b) 0.1–100, (c) 100–200, (d) 200–300 and (e) ⩾300.

To verify whether the spatial distribution of COVID-19 occurs randomly in space, we performed spatial autocorrelation analysis of crude incidence rates by calculating the univariate global Moran index. For that, we elaborated a spatial proximity matrix obtained by the contiguity criterion, with a significance level of 5%. This index ranges from −1 to +1 so that values close to zero indicate spatial randomness; values between 0 and +1 indicate positive spatial autocorrelation and, between −1 and 0, negative spatial autocorrelation [21].

The global Moran autocorrelation coefficient is based on the cross products of the deviations from the mean being calculated for the observations as follows:
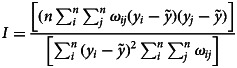
where *ω_ij_* is a contiguity matrix element (*ω*), *γ_i_* is the incidence rate of municipality *i*, *γ_j_* is the incidence rate of municipality *j*, 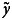 is the mean of the sample and the symbol *n* represents the total number of municipalities [22].

The local Moran index (or local index of spatial association; LISA) [13] was used to compare the value of each municipality with the surrounding municipalities and to verify the spatial dependence between them. In addition, to assess the local spatial grouping and to verify that the process stationarity hypothesis occurs locally, we obtained a measure of the association for each unit using the following equation [23]:
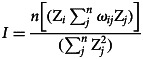
where 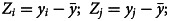 *ω_ij_* is the contiguous matrix element *ω*; *y_i_* is the incidence rate of municipality *i*; *y_j_* is the incidence rate of municipality *j*; 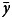 is the sample mean and the symbol *n* represents the total number of cities [22].

Subsequently, a scattering diagram was obtained with the following spatial quadrants: Q1 (high/high) and Q2 (low/low), which indicate municipalities with values similar to those of the surrounding ones, and represent areas of agreement with positive spatial association aggregates; Q3 (high/low) and Q4 (low/high) indicate municipalities with differing values and which represent transition areas with aggregates of negative spatial association [22]. The significant results were visually expressed on Moran maps.

### Prospective spatiotemporal cluster analysis

The prospective space–time scan statistic was performed to identify high-risk space–time clusters for transmission of COVID-19, using the Poisson probability distribution model [13, 24]. This analysis allows us to evaluate potential clusters that are still occurring at the end of the study period. We consider as active space–time clusters (present), those that are still occurring, that is, in activity [6]. Our null hypothesis (H0) is that the expected number of COVID-19 cases in each area is proportional to the size of its population and indicates a constant risk of infection. Although the alternative hypothesis (H1) is that the number of observed cases exceeds the expected number of cases derived from the null model [6].

We built the cluster analysis model with the following conditions: minimum aggregation time of 2 days, minimum of five cases, without overlapping of clusters, circular clusters, the maximum size of the spatial cluster of 10% of the population at risk and maximum size of the temporal cluster of 50% of the study period [6]. The primary cluster and secondary clusters were detected using the log-likelihood ratio test and represented on maps [11]. We also calculated the RRs of the occurrence of COVID-19, considering each municipality and agglomerates in relation to the surrounding areas. Results with *P*-value <0.05 using 999 Monte Carlo simulations were considered significant.

### Software

Microsoft Office Excel 2010 software was used for data tabulation and descriptive analysis; Joinpoint Regression Program v. 4.2.0 [25] for time trend analysis; QGis v. 3.4.11 (QGIS Development Team; Open Source Geospatial Foundation Project) for generating the choropletic maps [26]; TerraView v. 4.2.2 (Instituto Nacional de Pesquisas Espaciais, INPE, São José dos Campos, SP, Brazil) for the spatial analysis [27]; SaTScan™ 9.6 (Harvard Medical School, Boston and Information Management Service Inc., Silver Spring, MD, EUA) for spatiotemporal scanning and cluster analysis [28].

### Ethical considerations

This study used public-domain aggregate secondary data and followed national and international ethical recommendations, as well as the rules of the Helsinki Convention.

## Results

During the first 11 weeks, after the diagnosis of the first case, 113 951 cases of COVID-19 were confirmed in the states of the Northeast region of Brazil. As a result, the average incidence rate in that period was 199.73 cases per 100 000 inhabitants. In absolute percentage values, the state of Ceará had the highest number of registered cases of COVID-19, corresponding to 29.40% of the total cases and is considered as the epicentre of the epidemic in the Northeast region. Next are the states of Pernambuco (22.57%) and Maranhão (16.47%). The state of Piauí had the lowest proportion of cases (2.86%).

**Fig. 2.**
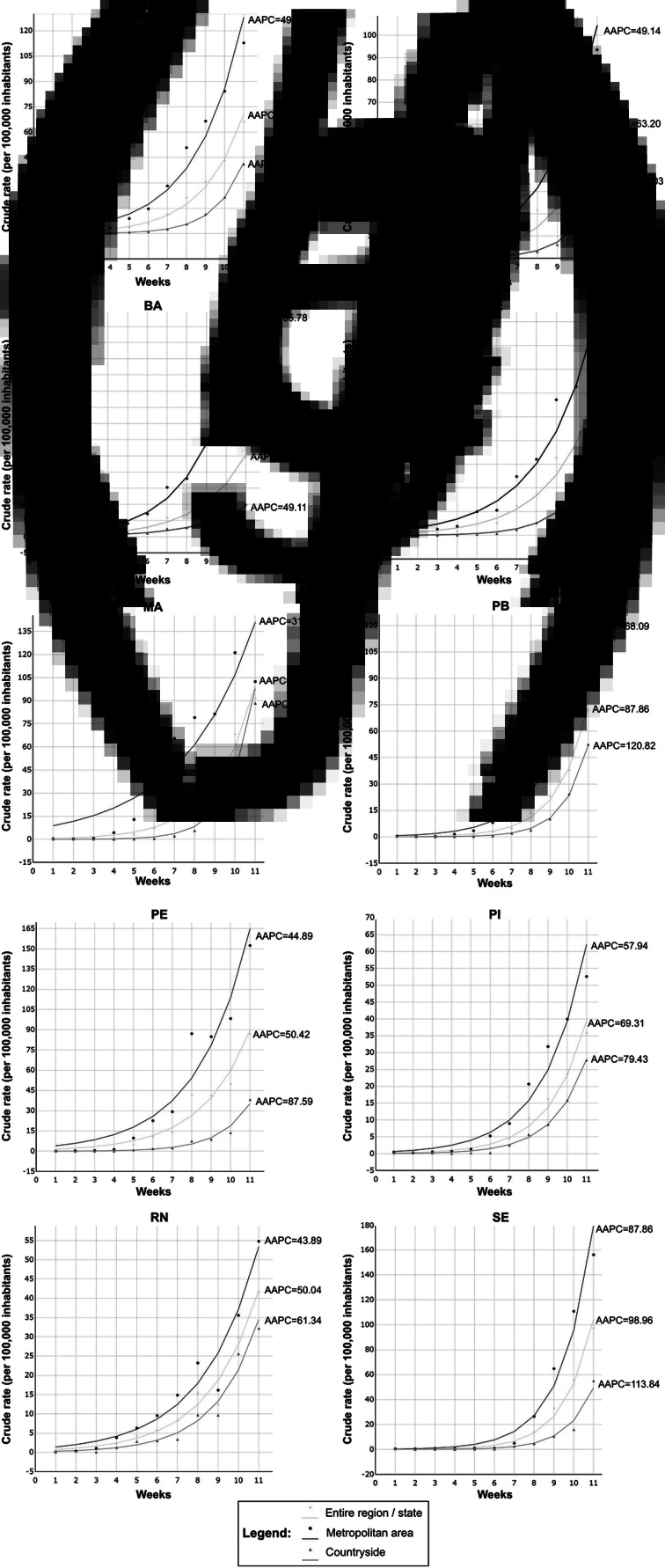
Weekly trends in incidence rates in the region, by state and by metropolitan and countryside areas of the states.

We carried out the time trend analysis according to the number of cases diagnosed per week (Table 1). In Figure 2A–J we present the weekly trends following the incidence rates in the region, by state, and by the metropolitan and countryside areas of the states. We observed an increasing trend in the crude incidence rate of the Northeast region of Brazil population, which presented an AAPC of 76.8 (95% CI 64.1 to 90.5; *P*-value <0.01; Fig. 2A). Similarly, increasing trends were observed in all states. However, the highest growth rates were observed in the states of Alagoas (AAPC, 134.1; 95% CI 91.2 to 186.7; *P*-value <0.01; Fig. 2B) and Sergipe (AAPC, 128.5; 95% CI 89 to 176.2; *P*-value <0.01; Fig. 2J). Although the state of Ceará had the highest percentage of cases in the region, the AAPC was the lowest recorded in the study period (AAPC, 61.0; 95% CI 46.7 to 76.8; *P*-value <0.01; Table 1). Importantly, the largest AAPCs were recorded in the countryside when compared to AAPCs in metropolitan areas of all nine states.

**Table 1.** Time trends of incidence rates of COVID-19 in the Northeast region by states

State/region	APC (95% CI)		
	Entire state/region	Metropolitan area	Countryside
NE	Segment 1: 1st to 8th 92.5 (69.2 to 119.1)*	Segment 1: 1st to 8th 90.7 (69.0 to 115.2)*	Segment 1: 1st to 11th 97.4 (90.9 to 104.0)*
	Segment 2: 8th to 11th 45.0 (34.7 to 56.0)*	Segment 2: 8th to 11th 29.1 (19.5 to 39.5)*	
AL	Segment 1: 1st to 8th 189.4 (102.5 to 313.5)*	Segment 1: 1st to 8th 191.9 (116.0 to 294.4)*	Segment 1: 1st to 5th −13.2 (−64.2 to 110.5)
	Segment 2: 8th to 11th 42.8 (25.9 to 62.0)*	Segment 2: 8th to 11th 24.9 (10.9 to 40.7)*	Segment 2: 5th to 11th 148.5 (117.3 to 184.1)*
BA	Segment 1: 1st to 5th 120.3 (16.6 to 316.0)*	Segment 1: 1st to 7th 82.5 (49.0 to 123.4)*	Segment 1: 1st to 11th 49.1 (38.6 to 60.4)*
	Segment 2: 5th to 11th 49.8 (42.4 to 57.6)*	Segment 2: 7th to 11th 47.7 (37.4 to 58.8)*	
CE	Segment 1: 1st to 9th 70.9 (49.8 to 95.0)*	Segment 1: 1st to 9th 64.2 (41.7 to 90.2)*	Segment 1: 1st to 9th 132.6 (105.3 to 163.5)*
	Segment 2: 9th to 11th 26.9 (−1.0 to 62.6)	Segment 2: 9th to 11th 16.2 (−15.3 to 59.5)	Segment 1: 9th to 11th 58.0 (39.6 to 78.8)*
MA	Segment 1: 1st to 7th 138.5 (32.8 to 328.4)*	Segment 1: 1st to 7th 147.0 (58.6 to 284.8)*	Segment 1: 1st to 11th 128.2 (97.8 to 163.3)*
	Segment 2: 7th to 11th 57.7 (43.1 to 73.9)*	Segment 2: 7th to 11th 13.6 (0.5 to 28.4)*	
PB	Segment 1: 1st to 8th 125.0 (82.9 to 176.7)	Segment 1: 1st to 8th 114.2 (66.7 to 175.3)*	Segment 1: 1st to 3rd −47.7 (−92.5 to 266.8)
	Segment 2: 8th to 11th 76.9 (64.3 to 90.4)*	Segment 2: 8th to 11th 51.3 (34.1 to 70.6)*	Segment 2: 3rd to 11th 132.8 (120.0 to 146.4)*
PE	Segment 1: 1st to 8th 108.6 (39.3 to 212.4)*	Segment 1: 1st to 8th 111.3 (45.0 to 207.8)*	Segment 1: 1st to 11th 87.6 (68.8 to 108.5)*
	Segment 2: 8th to 11th 30.9 (8.5 to 57.9)*	Segment 2: 8th to 11th 22.4 (1.8 to 47.0)*	
PI	Segment 1: 1st to 8th 137.0 (116.7 to 159.2)*	Segment 1: 1st to 8th 106.6 (71.9 to 148.3)*	Segment 1: 1st to 11th 79.4 (64.6 to 95.6)*
	Segment 2: 8th to 11th 49.7 (43.8 to 55.8)*	Segment 2: 8th to 11th 36.2 (20.7 to 53.6)*	
RN	Segment 1: 1st to 11th 50.0 (39.3 to 61.7)*	Segment 1: 1st to 11th 43.9 (32.2 to 56.6)*	Segment 1: 1st to 11th 61.3 (48.2 to 75.6)*
SE	Segment 1: 1st to 9th 144.6 (83.3 to 226.3)*	Segment 1: 1st to 11th 87.9 (61.9 to 118.0)*	Segment 1: 1st to 11th 113.8 (83.9 to 148.6)*
	*Segment 1:* 9th to 11th 74.0 (32.5 to 128.4)*		

AL, Alagoas; BA, Bahia; CE, Ceará; MA, Maranhão; PB, Paraíba; PE, Pernambuco; PI, Piauí; RN, Rio Grande do Norte; SE, Sergipe.

*P-value <0.05.

**Fig. 3.**
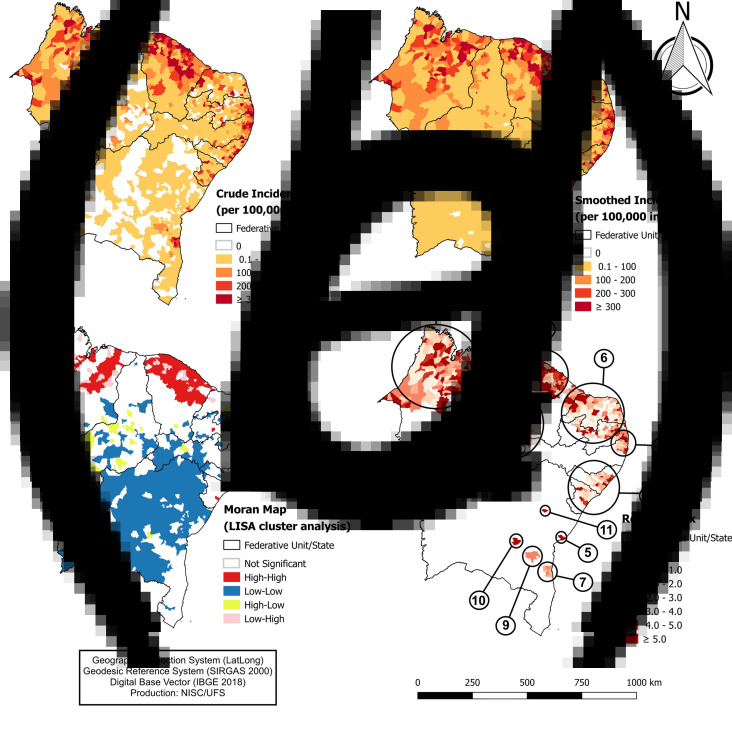
Spatial analysis of COVID-19 among municipalities in the Northeast region of Brazil. (A) Gross incidence rate; (B) smoothed incidence rate; (C) Moran map (LISA cluster) and (D) RR (spatial modelling by prospective scanning).

Subsequently, to identify areas with a higher concentration of COVID-19 cases, we assessed the spatial distribution of the disease among Northeast region of Brazil municipalities (Fig. 3A–D). We observed that cases of COVID-19 were widely distributed in the region, with records in 76.76% (*n* = 1378) of the municipalities (Fig. 3A). Interestingly, the cities with the highest numbers of confirmed cases were Fortaleza (capital of CE; *n* = 19 270) and Recife (capital of PE; *n* = 12 523). On the other hand, Salvador (state of BA) is the most populous capital of the Northeast region of Brazil, however, it presented less than half the number of cases (*n* = 7118) than Fortaleza. The state capitals were responsible for 57 959 cases, equivalent to 50.86% of all cases.

We also identified that 23.17% of the municipalities (*n* = 416) did not register cases of the disease. However, when considering smoothed rates, this percentage was reduced to 0.61% (*n* = 11; Fig. 3B). Even with spatial smoothing techniques, the highest incidence rates (areas of greatest risk of COVID-19) were concentrated in the coastal strip of the Northeast region of Brazil, where the metropolitan areas of the states are located. Similarly, significant spatial autocorrelation (high/high; *I* = 0.373; *P* = 0.001; Fig. 3C) was reported in the metropolitan areas and 178 municipalities considered a priority, especially in the states of CE and MA.

Next, we performed the prospective space–time scan statistics (Table 2) and identified 11 spatiotemporal clusters of COVID-19 cases (Fig. 3D). The primary cluster (cluster number 1) included 70 municipalities, all from the state of Ceará, and the largest number of cases (22 007), in the period from 4 to 22 May. The crude incidence rate in this cluster was 240.98 cases per 100 000 inhabitants and an RR of 9.64. Of the total clusters identified, five were in the state of Bahia, where the cluster with the highest RR was reported (cluster 10; RR = 19.46).

**Table 2.** Emerging space–time clusters of COVID-19 from 6 March to 22 May 2020

Cluster	Duration (days)	Number of municipalities	States	Observed	Expected	RR	LLR	Municipalities with RR > 1
1	4 May to 22 May	70	Ceará	22 007	2760	9.64	28 212	67
2	26 Apr to 22 May	52	Pernambuco, Paraíba	21 929	3922	6.68	21 296	45
3	5 May to 22 May	159	Maranhão	13 058	2610	5.52	11 079	125
4	7 May to 22 May	105	Alagoas, Sergipe	6506	1684	4.04	4074.4	57
5	21 May to 22 May	1	Bahia	1787	147	12.33	2834.3	1
6	12 May to 22 May	147	Rio Grande do Norte, Paraíba, Ceará	4894	1561	3.23	2307.8	147
7	23 Apr to 22 May	7	Bahia	1109	338	3.3	548.3	7
8	11 May to 22 May	157	Maranhão, Piauí, Ceará, Pernambuco	1756	875	2.02	345	48
9	9 May to 22 May	1	Bahia	182	55	3.26	88.77	1
10	21 May to 22 May	1	Bahia	16	0.82	19.46	32.31	1
11	20 May to 22 May	1	Bahia	17	2.19	7.77	20.04	1

RR, relative risk for the cluster compared with the rest of the region; LLR, log-likelihood ratio.

## Discussion

This study analysed the incidence and spatial distribution, and identified the occurrence of risk clusters for SARS-CoV-2 infection in municipalities from northeast Brazil. We reported herein that COVID-19 is a serious public health problem in the Northeast region of Brazil, which lead the ranking of higher incidence and mortality rates of Brazil [11]. In fact, several studies have been investigating the spatial dynamics of the disease, but few of them have applied the integration of methods of time trends, spatial clusters and prospective spatiotemporal clusters to analyse the COVID-19 pandemic [4, 8, 13, 29]. Taken together, our results demonstrate the exponential growth of COVID-19 in the Northeast region of Brazil and the rapid spread of cases from metropolitan areas to countryside municipalities.

We observed an increasing temporal trend in all states of the Northeast region of Brazil. Importantly, the highest growth rates were observed in the states of Alagoas and Sergipe, whose AAPCs were even higher than those observed in the Northeast region. Conversely, we notice a centripetal dispersion of the COVID-19 cases on the states. Data from our study demonstrated either an expansion process of the disease towards countryside municipalities, given that AAPCs of the countryside from almost all states (except for Bahia state) were superior when compared to AAPCs of entire region/states or metropolitan areas. These findings warn of the severity of dispersing cases and the projection of collapse in public health systems. Most municipalities in the interior of the states do not have hospitals with exclusive clinical assistance for COVID-19. Expanding cases and increasing demand for clinical care, many patients in these municipalities will be referred to hospitals in metropolitan regions, which are already in a state of overcrowding.

The spatial distribution of COVID-19 revealed a wide distribution of the disease in all states, except in Bahia state, herein we observed low incidence rates or absence of cases in countryside municipalities. Interestingly, when we analysed the smoothed rates, a dispersion in this area was also evidenced, since this statistical method considers the proximity of neighbours with confirmed cases. Our results showed clustering of highest incidence rates located in the Salvador metropolitan area like other Brazilian regions. The state of Bahia (and the capital Salvador) is the most populated areas in the Northeast region. However, we observed a distinct epidemiological panorama, with lower incidence and time trend (AAPCs) less than other states and metropolitan areas. We hypothesised that measures to combat the disease were implemented early in the pandemic, such as the blockade of state highways, which may have reduced the spread of the disease among countryside municipalities. It may also indicate diagnosis failures due to low population testing. However, we emphasise that further studies are required to understand the dynamics of the disease in the state of Bahia [30].

Tourism, economic networks and social mobility are important factors to better understanding of the disease progression in different territories [31]. In Wuhan, China, social mobility was associated with high transmission of SARS-CoV-2, and social distancing policies were effective at controlling the epidemic [32]. Thus, we recommend the strengthening of these measures considering the increasing trends of COVID-19 in the Northeast region. This region is the most-searched travel destination of Brazil, especially Ceará state. Additionally, Fortaleza (capital of Ceará state) is the nearest city from Europe and has heavy air traffic (national and international), which probably explains the highest incidence of COVID-19 in the Northeast region of Brazil.

The spatiotemporal analysis enabled us to visualise the heterogeneous distribution of COVID-19 and identify spatial dependence and priority areas. Studies using these techniques supported the understanding of disease dissemination towards neighbouring areas in China [4, 7, 29, 33]. Besides, detection of high-risk spatiotemporal clusters can guide the decision making related to the implementation of more strict policies [6]. Furthermore, spatial modelling can assist and guide the implementation of control measures to reduce or prevent the spread of the virus [4, 7, 33].

We also highlight the limitations of the study, which include the use of secondary data reported by health departments. In some records (*n* = 1519) we did not find information on the location of the cases. In addition, states have adopted testing policies with different criteria since the beginning of virus circulation in the country. We also point out that massive testing policies have not been implemented in Brazil, with symptomatic cases and/or those seeking health services being strictly notified. This may indicate, therefore, that the number of COVID-19 cases in Brazil is underreported. Despite the limitations found here, the analyses were not compromised, and our findings bring relevant data and support for decision-making and the formulation of new public policies to face the epidemic in Brazil.

We emphasise that the incorporation of geostatistics techniques was able to highlight areas of risk for the occurrence of COVID-19. Additionally, we identified priority regions in Northeast region of Brazil to mitigate the impacts on health and the economy, as well as to assist in the allocation of resources and mobility restriction measures. However, for health monitoring of COVID-19, new studies are required, which may include prospective spatiotemporal modelling, addressing different socio-demographic strata and analysing socioeconomic indicators of the regions.

## Conclusion

Altogether, our results showed that the epidemic of COVID-19 is growing exponentially in all states of the Northeast region, with priority clusters mainly in the states of Ceará and Maranhão. The results also demonstrate the dispersion of cases to countryside municipalities of the states. COVID-19 represents a serious public health problem, and its impact may be greater, considering the interiorisation process and its growing expansion to more vulnerable areas and without exclusive clinical care for the disease. The dynamics of transmission and the repercussions of COVID-19 in the Northeast region have not yet been fully elucidated and require further studies.

## Data availability statement

The data that support the findings of this study will be available on request and permission of via e-mail from the corresponding author.
